# Structural Consequences of Antigenic Variants of Human A/H3N2 Influenza Viruses

**DOI:** 10.3390/v15041008

**Published:** 2023-04-19

**Authors:** David Francis Burke

**Affiliations:** European Molecular Biology Laboratory, European Bioinformatics Institute (EMBL-EBI), Wellcome Genome Campus, Hinxton, Cambridgeshire CB10 1SD, UK; dburke@ebi.ac.uk

**Keywords:** influenza, antigenic, protein structure, hemagglutinin

## Abstract

The genetic basis of antigenic drift of human A/H3N2 influenza virus is crucial to understanding the constraints of influenza evolution and determinants of vaccine escape. Amino acid changes at only seven positions near the receptor binding site of the surface hemagglutinin protein have been shown to be responsible for the major antigenic changes for over forty years. Experimental structures of HA are now available for the majority of the observed antigenic clusters of A/H3N2. An analysis of the HA structures of these viruses reveals the likely consequences of these mutations on the structure of HA and thus, provides a structural basis for the antigenic changes seen in human influenza viruses.

## 1. Introduction

**Figure 1:**
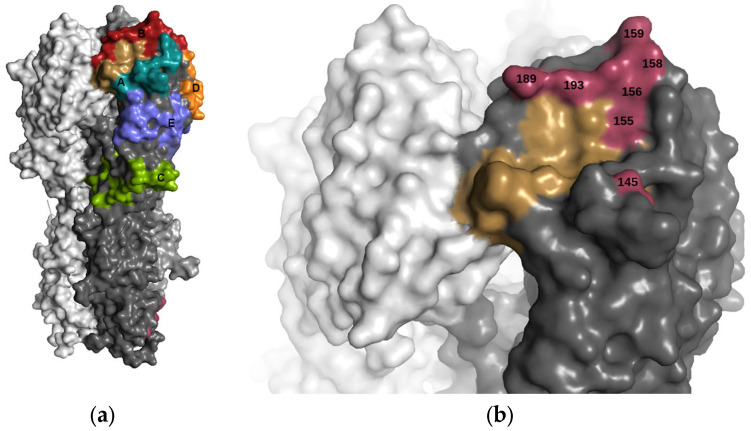
Structure of the trimer of the haemagglutinin protein of A/H3N2 influenza virus. (**a**) The five antigenic sites A–E are coloured cyan, red, green, orange and blue, respectively. The receptor binding site is coloured in gold. (**b**) Close-up of the receptor binding site of HA (coloured in gold). The seven amino acid positions at which mutations have been shown to be sufficient for human H3 antigenic evolution are highlighted.

The hemagglutinin (HA) gene segment of influenza virus codes for a glycoprotein which is expressed as an extended trimer on the surface of the viral capsid and is the major target of the antibody response to influenza virus infections. The protein is cleaved into two polypeptide chains, HA1 and HA2, which are held together by a series of di-sulphide bonds. HA attaches to cells in the upper respiratory tract by binding to a class of sialic acid-containing glycans in its receptor binding site (RBS) located at the membrane distal end of the trimer. Influenza viruses can escape antibody neutralisation by the accumulation of mutations in HA. Early work based on monoclonal antibodies defined five antigenic sites (A–E) on the globular head of HA as main targets for these antibodies (Figure 1) [1,2]. Smith showed that between the introduction of the A/H3N2 virus in humans in 1968 and 2003, there were eleven clusters of viruses with distinct antigenic properties, each of which was rapidly replaced by successive clusters [3]. It was previously thought that viruses needed four or more amino acid substitutions located in at least two of these sites to change its antigenicity sufficiently to form a new cluster. Indeed, there were sixty-seven amino acid substitutions at fifty-four positions associated with the A/H3N2 antigenic clusters and almost all of these were in the five previously defined antigenic sites [3]. However, Koel and colleagues reported the genetic basis of the major antigenic evolution for this same dataset [4] and found that substitutions at only seven amino acid locations are responsible for the major antigenic changes in these viruses. Surprisingly, a single amino acid substitution was sufficient to explain the antigenic differences between seven of these cluster transitions; two clusters were caused by two substitutions, and the remaining cluster by three substitutions. Fonville later showed that viruses circulating in subsequent genetic clades (CAL04, WI05, BR07, and PE09) also formed distinct antigenic clusters [5]. Although the genetic determinants of these antigenic clusters has not yet been fully established, differences in these same seven sites as described by Koel are seen [3]. In contrast, the genetic clades PE09 and 3C.1 have been shown to be antigenically similar [5,6] with identical amino acids in these seven positions.

The mutations responsible for transitions between antigenic clusters occurred at positions 145, 155, 156, 158, 159, 189 and 193 and are located on the exposed surface of the HA, peripheral to the receptor binding site (Figure 1). Two of them, at positions 189 and 193, lie close to each other on the solvent exposed face of an alpha-helix in antigenic site B which runs along the side of the RBS proximal to the trimer interface. Residues 155, 156, 158 and 159 are located in a loop adjacent to this and can interact with the nearest residues on the helix including 193. The final amino acid, at position 145, is located in a solvent exposed loop at the distal side of the RBS in antigenic site A.

These sites are also likely to be important for more recent genetic clades. Chambers and colleagues showed that viruses with the Phe^159^Ser mutation had diminished antibody binding to ferret antibodies from clade 3C.1 [7]. Li et al. also demonstrated that viruses carrying either Phe^159^Ser and Asn^225^Asp, or Phe^159^Tyr and Asn^225^Asp were among the most frequent escape mutants from a 3C.1 virus when selected by human antisera [8]. The recent antigenic evolution of the genetic lineages 3C.2A and 3C.3A are correlated with the mutations Phe^159^Tyr and Phe^159^Ser, respectively. However, the analysis by Jorquera suggested that the mutation Phe^159^Tyr is unlikely to be the major cause of the antigenic change for the 3C.2A clade and most likely it is the introduction of a potential N-linked glycosylation site at the adjacent position 158 [6].

The importance of these amino acid positions is not restricted to human A/H3N2 viruses alone. It has also been shown that changes at or close to these seven positions cause major antigenic differences in human A/H1pdm [9] and A/H2N2 viruses [10] as well as avian A/H5N1 [11], equine A/H3N8 [12] and swine A/H1 [13] and A/H3 viruses [14]. Doud showed that for A/H1N1, single amino-acid mutations can escape both strain-specific antibodies as well as broadly neutralizing monoclonal antibody targeting to the receptor binding pocket [15]. Mutations seen at sites 156, 158 and 193 had a strong effect on antibody escape.

For A/H3N2 viruses, there is an almost complete understanding of the genetic changes responsible for the major antigenic differences over the last 50 years. For all bar three of these antigenic clusters, there is also an experimentally determined structure of a representative virus. In this manuscript, the observed structural features associated with the mutations responsible for the key antigenic differences seen throughout the evolution of A/H3N2 are examined to try to understand a structural mechanism for the observed antigenic changes.

## 2. Materials and Methods

### Structures of Cluster—Representative Strains

Experimentally derived structures of HA have now been described for twenty distinct human seasonal A/H3N2 viruses (Table 1) representing fifteen antigenic clusters. In addition to those defined by Smith et al. [3], structures are available from viruses which belong to the recent genetic clades CAL04, WI05, BR07, VI09, 3C.1, 3C.2A and 3C.3A. Structures for viruses in three antigenic clusters (BE89; BE92; and WU95) are yet to be determined. For these cases, a representative strain of the antigenic cluster as defined in Smith [3] was modelled based on structures available using MODELLER [16]. Exploration of alternative side chain conformations for relevant cluster-transition amino acid substitutions was performed by using a rotamer search algorithm [17] to predict the rotamer with the lowest predicted energy.

## 3. Results

### 3.1. Antigenic Differences Explained by Changes in Biophysical Properties

As expected, the amino acid composition at these key positions in HA seems limited by their exposed nature, Phe being the only hydrophobic amino acid frequently observed (Table 1). The necessity to both maintain a functional HA structure capable of binding cell-surface glycans and to escape neutralizing antibodies may have restricted the range of available amino acids. Analysis of the available structures show that none of the mutations result in significant movements in the backbone and their effect must be explained purely in terms of the change in their biophysical properties of the sidechain. A brief summary of the biophysical properties of amino acids is given in Section 2.1 of the Supplementary Materials and Table S1. Importantly, amino acids within the same biophysical grouping will often form similar interactions and are frequently substituted for each other, usually with minor disruption to the structure of the protein or its interactions [18]. Indeed, this observation is the basis of homology modelling of protein structures [19]. Conversely, changing an amino acid to one in a different class is more likely to alter the structure of the protein and affect its interactions. It has been shown that single amino acid changes are sufficient to alter the structure of local regions of proteins and can disrupt protein–protein interactions leading to disease [20].

Indeed, almost all the substitutions responsible for the cluster transitions substantially changed the biophysical properties of the amino acids involved (Table 2). Eleven of the twenty-one cluster–transition substitutions resulted in substantial changes to the size of the sidechain and twelve involved a change in the charge of the sidechain. Of the charge-changing mutations, six have small changes of the size of the amino acid sidechain. Interestingly, the overall charge in these positions over the whole period was zero, excluding cluster transition 6, which was an evolutionary dead-end. A simple mechanism based on changes to the biophysical properties of solvent-exposed amino acids on HA can be proposed to explain most of the antigenic differences. Commonly, this would involve a change to a different biophysical grouping. Thus, an increase in size of the sidechain would produce a protrusion in the solvent-exposed surface of the HA rim. Similarly, a change in the charge of the sidechain would alter the electrostatic charge at the protein surface. Such substitutions will lead to a change in the ability of individual antibodies to form optimal protein–protein interactions with HA, reducing their binding affinity, and thus, cause a change in the antigenic properties of the virus.

### 3.2. Magnitude of Antigenic Change: Neighbouring Interactions and Conformational Dynamics

Substitutions with changes to their biophysical properties cause an antigenic change equivalent to between a two-fold and five-fold decrease in the assay titre. (Table 2). It has previously been shown that the degree of an antigenic effect is largely consistent in both directions between antigenic clusters [4]. However, it has previously been shown that secondary mutations can affect antigenic effect of binding to monoclonals [21]. As detailed below, some of the mutations observed give rise to significantly larger changes and this simple hypothesis of change of biophysical properties is not sufficient.

The substitution responsible for cluster transition 7 (SI87 → BE92, Glu^156^Lys) has the largest antigenic change of any of the cluster transitions studied resulting in an antigenic distance equivalent to an average decrease of over 64-fold decrease of titres to relevant antibodies. This substitution causes a reversal of charge, with only a small increase in sidechain volume. Although this mutation is expected to affect the surface properties of the protein, the antigenic effect is more than twice as large as average. Indeed, ten years earlier, the reverse mutation, Lys^156^Glu (cluster transition 4), was observed, which caused a five-fold smaller antigenic change. It is also three-fold larger than the identical mutation at a nearby antigenically important site, position 158, which occurred less than five years later.

**Figure 2:**
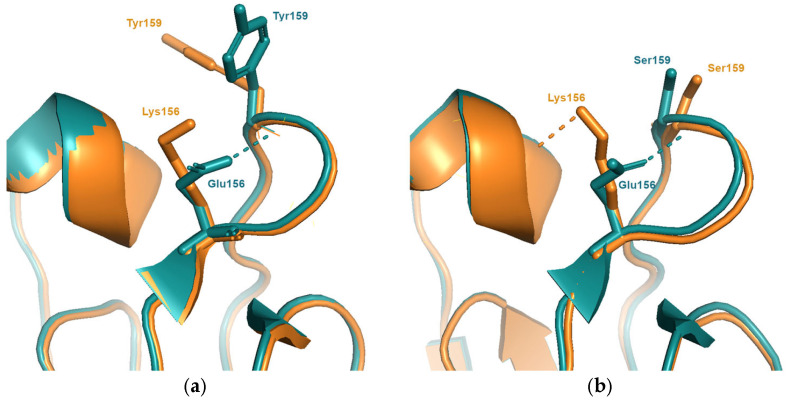
(**a**) The substitution Glu^156^Lys which is responsible for the antigenic change between viruses in the SI87(blue) and BE92(orange) clusters. Compared to the structure of the SI87 representative (A/Sichuan/2/1987; PDB entry 6pyp), the model of the BE92 representative (A/Netherlands/179/1993) is predicted to induce an additional structural rearrangement involving the sidechain of Tyr^159^. (**b**) The same substitution occurs between the earlier clusters BK79(blue) and TX77(orange). The structures of A/Texas/1/1977(PDB entry 6mxu) and A/Philippines/2/1982(PDB entry 6mym) show that, due to the presence of a smaller amino acid at position 159 (Ser) the mutation Glu^156^Lys does not give rise to additional structural changes thus resulting in a much smaller antigenic change.

Analysis of models for representative viruses of the BE92 cluster suggest a possible mechanism. The sidechain of Tyr^159^, seen in viruses in the SI87 cluster, is predicted to adopt the same conformation seen in all experimental structures with a bulky aromatic amino acid at position 159 (Figure 2). The Glu^156^Lys mutation would cause a clash between Lys^156^ and Tyr^159^, forcing the latter to adopt an alternative sidechain conformation. This additional structural change would account for the increased antigenic effect. Viruses in the earlier TX77 or BK79 clusters (cluster transition 4) both contain the smaller amino acid Ser^159^. Structures available for the TX77 and BK79 viruses shows that no rearrangements of the Ser^159^ sidechain are required to accommodate the Lys^159^Glu substitution and thus, there are no additional antigenic effects.

**Figure 3:**
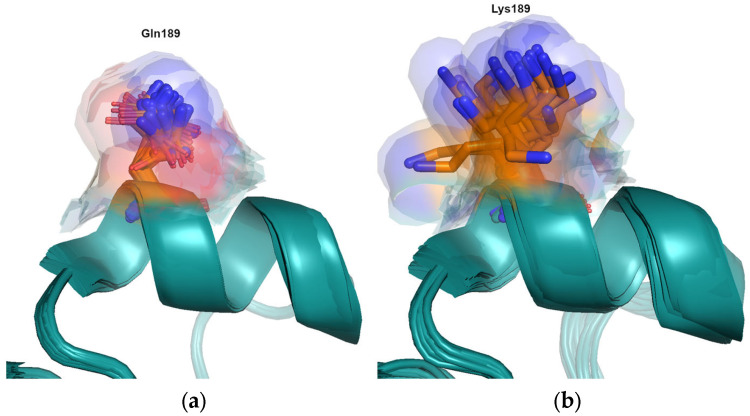
(**a**) Structures are available for viruses from the HK68(A/Hong-Kong/1/1968 PDB entry 4fnk) and EN72(A/Port-Chalmers/1/1973 PDB 4we5) clusters which contain Gln^189^ and show little conformational variability. (**b**) Many experimental structures are available for viruses which contain Lys^189^. Structures from the clusters VI75 (A/Victoria/3/1975; PDB entry 4gms), TX77 (A/Texas/1/1977; PDB entry 6mxu), BK79 (A/Netherlands/209/1980; PDB entry 6n08; A/Philippines/2/1982; PDB entry 6mym),VI09 (A/Victoria/361/2011; PDB entry 4we9), 3C.1 (A/Texas/50/2012; PDB entry 5w08; A/Singapore/H2011.447/2011; PDB entry 4o5n; A/Alberta/26/2012; PDB entry 4we7), 3C.2A (A/Michigan/15/2014; PDB entry 6bkp), and 3C.3A (A/Switzerland/9715293/2013; PDB entry 6pdx) show a large difference in conformational flexibility.

Koel showed that the mutation Gln^189^Lys has the second largest antigenic distance of any of the substitutions tested in their study [4]. Although there is a change in charge, the difference in length or volume of the sidechain is not large. The magnitude of this antigenic change is not fully explained by the changes in biophysical properties. An analysis of the available structures suggests that the conformational dynamics of the sidechains may also play a role. Lysine is the most inherently flexible sidechain of all the amino acids [21] and is significantly more likely to possess alternative sidechain conformation as Gln [22,23]. There are many structures available from viruses in the HK68 cluster, which contains Gln^189^, and of viruses containing Lys^189^ from clusters VI75, TX77, BK79, PE09, 3C.1, 3C.2A and 3C.3A. As expected, lysine is seen to adopt many more conformations, spanning nearly 9 Å, compared to glutamine, which spans 5 Å, resulting in a larger excluded volume (Figure 3). This difference is expected to affect the entropic component of antibody binding.

**Figure 4:**
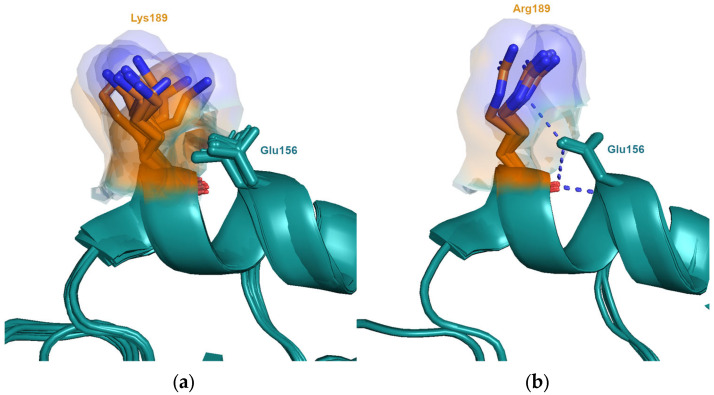
Interactions with neighbouring amino acids are shown to alter the allowed conformations sampled by the side-chains at position 189. (**a**) Experimental structures of viruses from the clusters VI75 (A/Victoria/3/1975; PDB entry 4gms), TX77 (A/Texas/1/1977, PDB entry 6mxu) and BK79 (A/Netherlands/209/1980, PDB entry 6n08, A/Philippines/2/1982, PDB entry 6mym) contain Lys^189^ and Asn^193^. In these structures, there is no interaction with neighbouring Asn^193^ and Lys^189^ is able to sample many conformations. (**b**) In the structure of the SI87 virus (A/Sichuan/2/1987; PDB entry 6p6p), the interaction of Arg^189^ with neighbouring Asn^193^ reduces the conformations observed.

Less than twelve years later, this position was again involved in a cluster transition. The substitution Lys^189^Arg (BK79 → SI87) was shown to have the largest individual antigenic effect of the three mutations found to be responsible for cluster transition 5 and the third largest overall [4]. This is unexpected since lysine and arginine have similar biophysical properties and are frequently observed to be substituted in the evolution of closely related protein sequences due to their ability to maintain interactions. Arginine also has a similar, albeit slightly reduced, inherent sidechain flexibility compared to lysine. However, the structure from the SI87 virus, A/Sichuan/2/1987, shows that the epsilon nitrogen of the sidechain of Arg^189^ is able to form an interaction with the sidechain of neighbouring Asn^193^, stabilising one particular conformation and thus restricting the observed conformations (Figure 4). The maximum distance of the distal nitrogen between the different conformations is 2.1 Å. Due to geometric constraints, Lys^189^ is unable to form this interaction resulting in many more conformations observed, the distal nitrogen differing by up to 8.4 Å. Thus, the differences in the ability to form this interaction significantly alters the excluded volume sampled by the two amino acids at this position. This restriction of conformational flexibility, and its resulting antigenic effect, is dependent on the genetic background of neighbouring positions, in this case, the amino acid at position 193.

### 3.3. Functional Equivalent Substitutions

**Figure 5:**
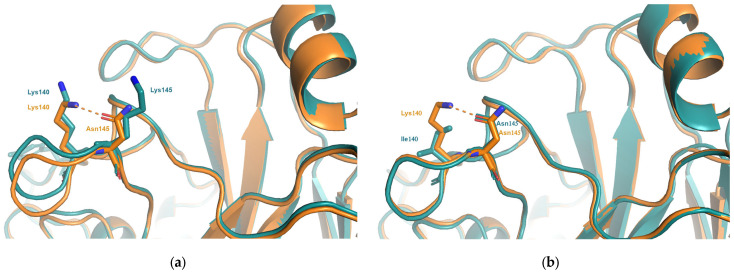
(**a**) In many structures, such as WI05 (A/Hong-Kong/4443/2005; PDB entry 2yp7, orange), Asn^145^ is seen to interact with Lys^140^. As seen in the structure of the FU02 virus, A/Wyoming/3/2003(PDB entry 6bko, blue), the substitution responsible for cluster transitions 6 and 8, Asn^145^Lys, disrupts this interaction resulting in changes in charge and a change in shape and volume at both positions 140 and 145. (**b**) The substitution Lys^140^Ile occurs between viruses in the WI05 and BR07 clusters. Comparing the experimental structures for WI05 (A/Hong-Kong/4443/2005; PDB entry 2yp7, orange) and BR07 (A/Brisbane/10/2007; PDB entry 6aop, blue) the interaction between 140 and 145 is lost and causes a change in volume at position 140.

The mutation Asn^145^Lys has been shown to be sufficient to explain the antigenic effect for two of the cluster transitions (SI87 → BE89 and BE92 → WU95). The reverse mutation, Lys^145^Asn, was shown to have the same antigenic effect in both the BE89 and BE92 clusters [4] and is also a cluster-determining substitution for the antigenic transition FU02 to Cal04. In the majority of the available structures, the sidechain of Asn^145^ is seen to interact with the sidechain of the neighbouring Lys^140^, stabilising the orientation of the loop (Figure 5). The Asn^145^Lys mutation not only changes the size and charge at position 145 but will remove this interaction, increasing the flexibility of the sidechain of Lys^140^ and likely altering the mobility of the loop. This also suggests that in viruses containing Asn^145^, mutating Lys^140^ would be functionally equivalent to the Asn^145^Lys substitution, resulting in a large change in charge and volume in this region and disrupting the intra-loop interaction between Lys^140^ and Asn^145^. Interestingly, the substitution Lys^140^Ile occurs between viruses the genetic clusters WI05 and BR07 (cluster transition 13) which were shown to have a four-fold difference in titre to many sera generated from viruses from 2003 to 2008 [5]. The genetics of this antigenic difference has yet to been fully established, but viruses in these clusters are identical in the seven positions identified by Koel, leaving the Lys^140^Ile mutation a likely candidate.

### 3.4. Role of N-Linked Glycosylation

The attachment of oligo-saccharides to the amide group of an asparagine sidechain can occur if the sequence motif Asn-X-Thr/Ser is present, where X is any amino acid except proline. Such carbohydrate attachments play important roles in the structural stability of many proteins on cell surfaces. The use of glycans to shield antibody epitopes on their respective surface glycoproteins has been observed in many viruses [24]. One of the best characterised glycan shields is that of the HIV-1 envelope glycoprotein, gp120, which contains up to 33 glycans per monomer [25]. The glycans can account for up to half of the total mass and cover up to 70% of the Env surface [26].

At its introduction into humans in 1968, the HA of influenza A/H3N2 virus contained only six N-linked glycosylation sequons. As it has evolved in humans, there has been a steady increase in sequons resulting in twice as many sites in recently circulating viruses (Table 3).

**Figure 6:**
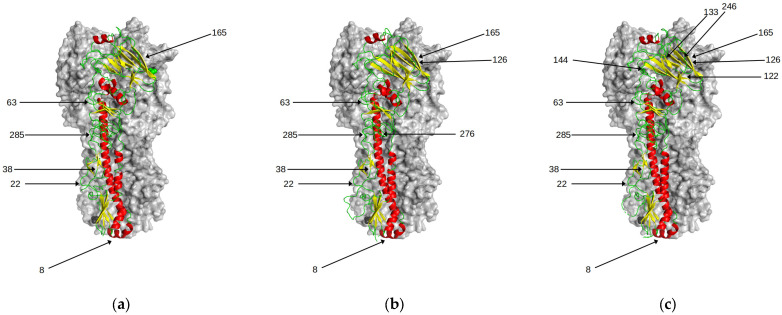

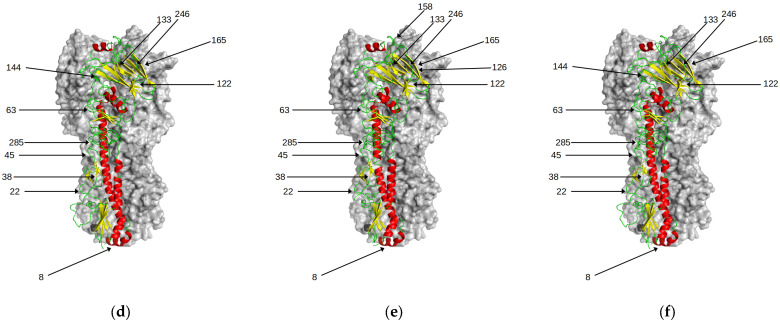
Predicted N-glycosylation sites in HA. There is a progressive increase over time in the number of N-glycosylation sites in HA. With the exception of position 158, these have little effect on antigenic properties of the viruses. Those sites are highlighted for the following genetic clusters (**a**) HK68 (**b**) BE92 (**c**) CAL04 (**d**) 3C.1 (**e**) 3C.2A1 (**f**) 3C.3A.

Analysing patterns of amino acid substitutions, Blackburne and colleagues showed that there was no correlation between changes in glycosylation and changes in antigenic clusters [27]. Interestingly, most of these newer sites have been in the sialic-acid binding region near the head of HA, a major target of the antibody responses (Figure 6). An analysis by Altman and colleagues suggests that H3 viruses acquire a new glycan site every five to seven years but are limited to a maximum of seven sites in the head region [28]. Das et al. showed that the introduction of N-glycans close to the RBS reduces binding to cell surface glycans [29]. Thus, this limits the use of excessive glycosylation of HA as a means of immune escape for the influenza virus, since it reduces overall avidity.

The introduction of these sites was shown to have little effect on the antigenicity of polyclonal ferret sera in the first 30 years of A/H3N2 evolution in humans [4]. For all bar one, none of the cluster-changing transitions involve a change in glycosylation site. The only exception is viruses belonging to the recent genetic clade 3C.2A which have seen an introduction of a sequon at the antigenically important position 158. This genetic change has been shown to be the key molecular determinant of the antigenic distancing of clade 3C.2A from clade 3C.1 [6]. Egg adapted vaccine strains derived from these viruses lose this seqon [30], potentially altering their antigenic profile. Future evolution of A/H3N2 may increasingly use glycan shielding in other regions of HA to evade acquired antibody immunity although the effect on viral avidity needs to also be considered.

## 4. Discussion

The evolution and conservation of amino acids in the head region of HA is governed by a balance of often competing influences. The overall fitness and viability of a mutant virus is a combination of at least three factors, namely a reduction of host antibody binding; protein structural stability; and the ability to bind to cell-surface glycans. The former will be time-dependent relative to the prevailing host immunity (the position in an antigenic map) and where on the protein the antibodies have targeted. Hence a mutation which gains a fitness advantage with respect to avoiding host immunity via antibody binding can compensate for a loss of the structural stability or reduction of binding host-cell glycans induced by that mutation. Subsequent mutations can help recover the overall fitness of the virus.

Most of the antigenic differences seen in human A/H3N2 influenza viruses can be explained based on simple changes in the biophysical properties of the amino acids involved. Changes in the biophysical properties of amino acids enable HA to reduce the affinity of antibodies close to the point of mutation and the cumulative addition of mutations involving changes in biophysical properties at more than one position can result in larger antigenic effects. This simple model for predicting the potential for antigenic change can be applied using genetic information alone with no consideration of structure.

However, mutations occur which greatly affect the antigenic properties of the virus even when the biophysical properties of amino acids are similar. These antigenic differences, over and above that expected from the changes in their amino acids properties, occur for at least three of the cluster–transition substitutions, (Glu^156^Lys; Gln^189^Lys; and Lys^189^Arg). A more sophisticated mechanism is required to understand the extent of the antigenic differences seen. For these mutations, either a re-arrangement of local structure is predicted in order to resolve clashes or differences in interactions with neighbouring amino acids cause a change in the flexibility of key antigenic sites, resulting in the differences to the antigenic binding surface. Thus, it is evident that the genetic background of the virus can abrogate or magnify the antigenic effect of a mutation. It is also likely that mutations in neighbouring positions also have the potential to cause large antigenic changes. The mutation Asn^140^Lys is predicted to give rise to identical structural rearrangements as Lys^145^Asn. However, other viral features such as structural stability or receptor binding need to be considered to assess whether these, and other such mutations, will become fixed within the viral population.

The need to account for allosteric effects caused by amino acids in close proximity to the seven highlighted by Koel and colleagues [4] complicates the prediction of antigenic changes based solely on the genetic information of a virus. The size of the antigenic difference is governed not only by changes in biophysical properties at these antigenically important sites but also by structural rearrangements of neighbouring residues induced by the mutation. The extent of these additional effects are dependent on the context of the genetic background and local structural features and will change as the virus evolves. The effect such antigenic changes have on glycan binding and overall structural stability of the virus also need to be considered. A detailed understanding of the structural mechanisms involved for each of these factors is critical for accurately predicting the future evolution and vaccine efficacy of A/H3N2 viruses.

## Figures and Tables

**Table 1:** Summary of the amino acids at regions shown to be important for antigenic cluster transitions are shown. Details of experimentally determined structures for viruses belonging to each antigenic cluster are given where available.

Cluster Name	Virus	PDB Entry	Resolution (Å)	Amino Acid Position							
				140	145	155	156	158	159	189	193
HK68	A/Aichi/2/1968	6e56	2.0	Lys	Ser	Thr	Lys	Gly	Ser	Gln	Ser
	A/HongKong/1/1968	4fnk	1.90								
	A/Northern-Territory/60/1968	6bkm	2.20								
EN72	A/Port-Chalmers/1/1973	4we5	2.10	Lys	Ser	Tyr	Lys	Gly	Ser	Gln	Asn
VI75	A/Victoria/3/1975	4gms	2.95	Lys	Ser	Tyr	Lys	Gly	Ser	Lys	Asn
TX77	A/Texas/1/1977	6mxu	1.85	Lys	Asn	Tyr	Lys	Glu	Ser	Lys	Asn
BK79	A/Netherlands/209/1980	6n08	1.92	Lys	Asn	Tyr	Glu	Glu	Ser	Lys	Asn
	A/Philipines/2/1982	6mym	2.45								
SI87	A/Sichuan/2/1987	6p6p	2.31	Lys	Asn	His	Glu	Glu	Tyr *	Arg	Asn
BE89	A/Netherlands/823/1992	Model	n/a	Lys	Lys	His	Glu	Asp	Tyr	Arg	Ser
BE92	A/Netherlands/179/1993	Model	n/a	Lys	Asn	His	Lys	Glu	Tyr	Ser	Ser
WU95	A/NetHiserlands/178/1995	Model	n/a	Lys	Lys	His	Lys	Arg	Tyr	Ser	Ser
SY97	A/Moscow/10/1999	6xpz	3.45	Lys	Lys	His	Gln	Lys ^†^	Tyr ^†^	Ser	Ser
FUO2	A/Wyoming/3/2003	6bko	1.65	Lys	Lys	Thr	His	Lys	Tyr	Ser	Ser
CAL04	A/Finland/486/2004	2yp2	1.90	Lys	Asn	Thr	His	Lys	Phe	Asn	Ser
WI05	A/Hong-Kong/4443/2005	2yp7	1.85	Lys	Asn	Thr	His	Lys	Phe	Asn	Phe
BR07	A/Brisbane/10/2007	6aop	2.30	Ile	Asn	Thr	His	Lys	Phe	Asn	Phe
VI09	A/Victoria/361/2011	4we9	2.20	Ile	Asn	Thr	His	Asn	Phe	Lys	Phe
3C.1	A/Texas/50/2012	5w08	2.60	Ile	Asn	Thr	His	Asn	Phe	Lys	Phe
	A/Singapore/H2011.447/2011	4o5n	1.75								
	A/Alberta/26/2012	4we7	2.50								
3C.2A	A/Michigan/15/2014	6bkp	2.05	Ile	Ser	Thr	His	Asn	Tyr	Lys	Phe
3C.3A	A/Switzerland/9715293/2013	6pdx	3.99	Ile ^‡^	Ser	Thr	His	Asn	Ser	Lys	Phe

* The sequence of A/Sichuan/2/1987 in the pdb entry 6pyp possesses Gln^159^ rather than the expected Tyr^159^. This mutation is often found in viruses which have a high number of passages in egg and cell lines. ^†^ The sequence of A/Moscow/10/1999 in the pdb entry 6xpz contains Glu^158^ and Asn^159^, whereas most available sequences of A/Moscow/10/1999 contain Lys^158^ and Tyr^159^. ^‡^ The sequence of A/Switzerland/9715293/2013 in the pdb entry 6pdx possesses Arg^140^ rather than Ile^140^. Arg^140^ is often found in sequences with a high number of passages in egg.

**Table 2:** Summary of the cluster–transition substitutions which have been shown to cause the largest antigenic change between subsequent antigenic clusters. The antigenic distance of a mutation is the distance between the relevant viruses in the 2-dimensional antigenic maps. The differences in charge, polarity, length and ASA are derived from the values shown in Table S1. These can be categorised into very small (yellow); small (saffron); medium (orange); and large (red).

Cluster	Cluster Transition	Citation	Mutation	Antigenic Distance	ΔCharge	ΔPolarity	ΔASA *	ΔLength	Size Category Change
1	HK68 → EN72	[4]	Thr^155^Tyr	2.6	0	−2.4	+85	+4.3	Small → Large
2	EN72 → VI75	[4]	Gln^189^Lys	4.1	+1	+0.8	+23	+2.3	Medium → Large
3	VI75 → TX77 *	[4]	Gly^158^Glu Asp^193^Asn	0.6	−1	+3.3	+138	+4.1	Very Small → Medium
				0.9	0	−1.4	−7	−1	Small → Small
4	TX77 → BK79	[4]	Lys^156^Glu	1.2	−2	+1.0	−29	−3.3	Large → Medium
5	BK79 → SI87	[4]	Ser^159^Tyr	1.6	0	−3	+107	+4.3	Small → Large
			Tyr^155^His	1.7	0	−4.2	−36	−1.9	Large → Medium
			Lys^189^Arg	3.1	0	−0.8	+29	−0.3	Large → Large
6	SI87 → BE89 *	[4]	Asn^145^Lys	3.1	+1	−0.3	+54	+3.8	Small → Large
7	SI87 → BE92	[4]	Glu^156^Lys	6.1	+2	−1.0	+29	+3.3	Medium → Large
8	BE92 → WU95	[4]	Asn^145^Lys	2.2	+1	−0.3	+54	+3.8	Small → Large
9	WU95 → SY97	[4]	Lys^156^Gln	2.2	−1	−0.8	−23	−2.3	Large → Medium
			Glu^158^Lys	2.1	+2	−1.0	+29	+3.3	Medium → Large
10	SY97 → FUO2	[4]	Gln^156^His	1.1	0	−0.1	+7	−0.5	Medium → Medium
11	FU02 → CAL04	[5]	Lys^145^Asn ^†^	3	−1	+0.3	−54	−3.8	Large → Small
12	CAL04 → WI05	[5]	Ser^193^Phe ^†^	2.5	0	−4.0	+95	+3.6	Very Small → Large
13	WI05 → BR07	[5]	Lys^140^Ile ^†^	2.5	−1	−6.1	−27	−2.8	Large → Medium
14	BR07 → VI09	[5]	Asn^189^Lys ^†^	3.2	+1	−0.3	+54	+3.8	Small → Large
			Lys^158^Asn ^†^		−1	+0.3	−54	−3.8	Large → Small
15	3C.1 → 3C.2A	[8]	Lys^160^Thr (N-Gly^158)^	2	0	−2.7	n/a	n/a	Large → Small
16	3C.1 → 3C.3A	[8]	Phe^159^Ser	6	0	+4.0	−95	−3.6	Large → Very Small

* The VI75 antigenic cluster was a dead-end lineage and strains in the TX77 antigenic cluster evolved from the EN72 antigenic cluster. An additional Asp^193^Asn mutation is required to take a VI75 mutant back to the root of EN72 and make it fully TX77-like. Similarly, BE89 was an evolutionary dead-end. Viruses from the SI87 cluster subsequently evolved into the BE92 cluster. ^†^ Clade specific differences are listed. Genetic link for these substitutions not yet established.

**Table 3:** N-glycosylation sites of HA A/H3N2 viruses. For each virus, the presence of a potential N-glycosylation site at each of the positions is shown as present (green) or absent (orange).

Virus	Genetic Clade	Position	8	22	38	45	63	81	122	126	133	144	158	165	246	276	285
		# Sites															
BI/16190/68	HK68	6															
BI/21793/72	EN72	6															
VI/3/75	VI75	7															
BI_2271_76	TX77	7															
NL/233/82	BK79	8															
A/Sichuan/2/87	SI87	8															
NL/823/92	BE89	8															
NL/179/93	BE92	9															
NL/178/95	WU95	8															
NL/301/99	SY97	11															
FU/411/02	FU02	11															
A/Finland/486/2004	CAL04	11															
A/HongKong/4443/2005	WI05	11															
A/Brisbane/10/2007	BR07	11															
A/Victoria/361/2011	VI09	12															
A/Texas/50/2012	3C.1	11															
A/Singapore/INFIMH-16-0019/2016	3C.2a1	12															
A/Switzerland/8060/2017	3C.2a2	12															
A/Cambodia/e0826360/2020	3C.2a1b.2a.1	12															
A/Darwin/9/2021	3C.2a1b.2a.2	11															
A/Switzerland/9715293/2013	3C.3a	11															
A/Kansas/14/2017	3C.3a	10															

## Data Availability

The structures presented in this study are openly available in the Protein Data Bank https://www.ebi.ac.uk/pdbe, accessed on 1 September 2022.

## Supplementary Materials

The following supporting information can be downloaded at: https://www.mdpi.com/article/10.3390/v15041008/s1, Table S1: Summary of biophysical properties of amino acids.
