# Supplementary material for: Structural Consequences of Antigenic Variants of Human A/H3N2 Influenza Viruses
Source: Viruses. 2023 Apr 19;15(4):1008. doi: 10.3390/v15041008 (PMC10144855; doi:10.3390/v15041008)
Supplement: Supplementary file 1 [file viruses-15-01008-s001.zip › viruses-2213086-supplementary.pdf]

S2. Materials and Methods

S2.1 Biophysical Properties of Amino Acids

Amino acids are commonly grouped according to similarities in various biophysical properties of the sidechain, notably the total charge and the polarity(the distribution of charge). This results in four groupings - those with a positively charged sidechain (Arg, His, Lys); a negatively charged sidechain (Asp, Glu); a polar, uncharged sidechain (Ser, Thr, Asn, Gln) or with a non-polar uncharged sidechain(Ala, Val, Ile, Leu, Met, Phe, Tyr, Trp)(Table S1). Additionally there are three amino acids which have unique properties - Cys, Gly and Pro(which is strictly an imino acid).

Table S1. Summary of biophysical properties of amino acids.

Amino Acid	Three Letter Code	Sidechain Accessible Surface Area (Å^2) *	Length (Å)	Net charge at pH 7.4	Polarity	Biophysical Property
Glycine	Gly	0	3.9	0	9	Non-polar
Alanine	Ala	67	5.5	0	8.1	Non-polar. β-branched sidechain
Serine	Ser	80	6.1	0	9.2	Polar. Hydrogen bonding possible
Threonine	Thr	102	6.1	0	8.6	Polar. Hydrogen bonding possible
Cysteine	Cys	104	6.4	0†	5.5	Non-polar. Sulphur atom able to form covalent bonds with the sulphur atom of another cysteine.
Proline	Pro	105	6.2	0	8	Non-polar. Very restricted conformation
Aspartic acid	Asp	106	6.5	-1	13	Hydrogen bonding possible
Asparagine	Asn	113	7.5	0	11.6	Polar. Hydrogen bonding possible
Valine	Val	117	7.0	0	5.9	Non-polar. β-branched sidechain
Leucine	Leu	137	8.5	0	4.9	Non-polar. β-branched sidechain
Glutamic acid	Glu	138	8.0	-1	12.3	Hydrogen bonding possible
Isoleucine	Ile	140	8.5	0	5.2	Non-polar. β-branched sidechain
Glutamine	Gln	144	9.0	0	10.5	Polar Hydrogen bonding possible
Histidine	His	151	8.5	0†	10.4	Aromatic
Methionine	Met	160	10.3	0	5.7	Non-polar. β -branched sidechain
Lysine	Lys	167	11.3	+1	11.3	Hydrogen bonding possible
Phenylalanine	Phe	175	9.7	0	5.2	Non-polar. Aromatic sidechain
Tyrosine	Tyr	187	10.4	0	6.2	Aromatic. Hydrogen bonding possible
Arginine	Arg	196	11.0	+1	10.5	Charged. Hydrogen bonding possible
Tryptophan	Trp	217	10.9	0	5.4	Non-polar. Aromatic

* The Solvent accessible surface area(ASA) of amino acid side chain for residue X in a Gly-X-Gly tripeptide is defined as the surface traced by the center of a sphere with the radius of a water molecule (0.15 mm) as it is rolled over the surface of the structure. This can be categorised into very small (yellow); small (saffron); medium (orange);large (red). †The imidazole sidechain of histidine has a pKa of approximately 6.0 so relatively small shifts in pH will change its average charge. At physiological pH(7.4), about 4% of histidines will be protonated and have a charge of +1. The pKa of cysteine is 8.3. At physiological pH, about 11% will be de-protonated and have a charge of -1.
